# Sticky tubes co-assembled by functionalised diphenylalanine and polydopamine nanoparticles form biocompatible antifouling coating†

**DOI:** 10.1039/d4ra08342c

**Published:** 2025-02-05

**Authors:** Subramaniyam Sivagnanam, Suman Nayak, Arpita Halder, Oindrilla Mukherjee, Abhijit Saha, Priyadip Das

**Affiliations:** a Department of Chemistry, SRM Institute of Science and Technology SRM Nagar, Potheri, Kattankulathur Tamil Nadu-603203 India priyadipcsmcri@gmail.com priyadip@srmist.edu.in abhijits@srmist.edu.in; b Department of Biotechnology, National Institute of Technology Durgapur West Bengal 713209 India

## Abstract

The persistent challenge of biofouling, driven by the accumulation of microorganisms and biological residues on surfaces, undermines operational efficiency and safety across multiple industries. Functionalized peptide based biocompatible and supramolecular coating can provide a substantial solution to this crucial issue. This present study describes the formation of polydopamine-comprised sticky tubes through the co-assembly of an antifouling peptide P1 (FF–PFB) and Polydopamine Nanoparticles (PDA NPs) with an adhesive catechol moiety. To overcome the synthetic complications associated with the attachment of adhesive l-DOPA or dopamine with antifouling peptides, we have employed a simple co-assembly strategy. These co-assembled sticky tubes form a stable, biocompatible coating on desired surfaces (glass and aluminium) and resist fouling. The design consists of a diphenylalanine-based antifouling peptide covalently coupled with pentafluoro benzaldehyde (PFB), which could self-assemble into a stable functional coating through the adhesive catechol moiety of PDA NPs. This functional coating effectively resists bacterial and protein adhesion. These sticky tubes coated desired surfaces (glass and aluminium) exhibit excellent antifouling activity against both tested Gram (+)ve (*S. aureus*) and Gram (−)ve (*E. coli*) bacterial strains. More importantly, this simple co-assembly and drop-coating method has significant promise, primarily attributed to its simplicity of operation, which reduces production costs and expands the potential for widespread commercialization. This study not only contributes to the fundamental understanding of the antifouling process but also offers a practical and sustainable solution to the challenges caused by biofouling. Our findings, achieved through the simple and effective co-assembly strategy with two different functional components, pave the way for developing promising antifouling materials with broad applications in industries where effective biofouling resistance is crucial.

## Abstract 2

Co-assembled sticky tubes obtained by co-assembling functionalized dipeptide and polydopamine nanoparticles form a stable, biocompatible coating on desired surfaces (glass and aluminium) and resist fouling.

## Graphical abstract

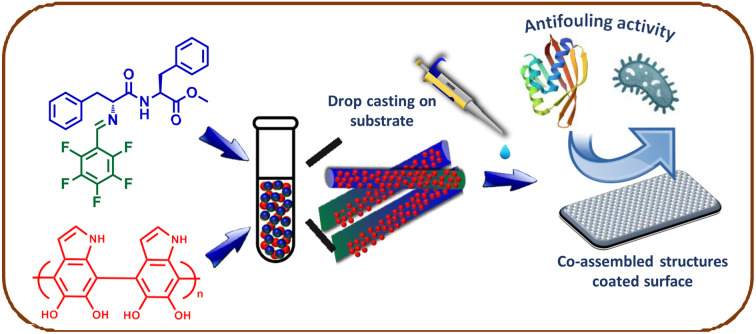

## Introduction

Biofilm formation has a significant impact on various sectors, which are associated with our daily lives, including medical implantation, biosensors, marine industries, water treatment, and food processing industries.^1^ The growing prevalence of bacterial infections in recent years has led to an urgent demand for antibacterial or antifouling surfaces. In general, nosocomial infection and bacterial fouling on various surfaces have been reported to be major causes of mortality, and they significantly increase the resistance of the bacteria toward antibiotics,^2^ which leads to medical complications and corresponding financial burdens. In the case of medical implants, the accumulation of proteins, specifically on biosensor surfaces, significantly affects their sensitivity and overall efficacy.^3^ In marine sectors, the formation of biofilms on the hulls of ships increases the mass of these vehicles and is responsible for additional fuel consumption.^4^

Among the several strategies, the employment of stable and biocompatible coating using antifouling materials has gained considerable attention due to their insignificant cytotoxicity, cost-effectiveness, and ease of operation. Smart antifouling materials are designed to inhibit the accumulation of unwanted biological organisms, such as algae, barnacles, proteins and bacteria on surfaces submerged in water,^5^ and an ideal antifouling coating should be cost-effective and expected to exhibit long-term stability, biocompatibility, and superior antifouling activity. However, many of the reported antifouling materials-based coatings failed to address the above-mentioned requirements. Therefore, there is an immense demand for the development of optimized approaches for the fabrication of long-term stable, biocompatible, and cost-effective smart antifouling coatings that sustain the biological environment without any adverse effects.

Three different strategies have been widely employed to inhibit the biofilm formation.^6^ The first strategy involves the use of biocides, which are antibacterial and specifically designed to abolish bacteria and other microorganisms. The second strategy focuses on repelling proteins and microorganisms to prevent the occurrence of biofilm formation. By impeding the initial adhesion of proteins and microorganisms, this approach aims to maintain surface cleanliness. The third strategy ascribes to the surfaces endowed with self-cleaning properties. Such surfaces facilitate the removal of attached microorganisms and ensure the resistance for their adhesion. Nature ingeniously combined these strategies to achieve effective antifouling mechanisms. In this regard, employment of naturally occurring biomolecules holds a significant promise with advantages of inherent biocompatibility, superior activity, and greater functionality compared to other reported synthetic compounds.^7^ Among the various biomolecules, peptides, specifically short peptides, have received substantial attention to develop smart antifouling materials since they are eco-friendly, biocompatible, and possess a natural propensity to self-assemble into well-ordered supramolecular architectures. The self-assembly of peptides represents a fundamental process in biomaterials science, where simple molecular building blocks spontaneously organize into complex and functional architectures. This process is driven by non-covalent interactions, including hydrogen bonding, π–π stacking, hydrophobic effects, electrostatic forces, and van der Waals interactions. Such versatility allows peptides to form diverse nanostructures of various morphologies and hydrogels/organogels, with tuneable properties tailored for specific applications.^8^ External factors such as pH, temperature, solvent polarity, and ionic strength significantly influence the self-assembly process, enabling precise control over the morphology and stability of the resulting materials. This adaptability makes peptide self-assembly a promising strategy for engineering advanced materials with hierarchical architectures.^9^ Furthermore, co-assembly of peptide with other functional molecules significantly enhance the employability of the co-assembled superstructures for various novel applications such as shape memory materials.^10^ Several reports show the ability of short peptides to form a functional antifouling coating that resists the biofouling process by reducing the initial non-specific adsorption of bacteria, proteins, and other microorganisms.^2,11–13^ Usually, peptide-based antifouling materials consist of three major components: (i) self-assembly moiety, (ii) antifouling unit, and (iii) adhesive unit.^14^ Most of the reported peptide-based smart antifouling materials comprised l-DOPA (l-3,4-dihydroxy phenylalanine) with catechol moiety as an adhesive/anchoring unit.^15–17^ This choice is preferred because DOPA is the key constituent of adhesive proteins of marine mussels (mussel foot proteins (mfps))^18,19^ and is able to adhere to different surfaces (metal, glass, Teflon)^20^ either through H-bonding or semi-covalent coordination bonding.^18^ The oxidized form of DOPA plays an important role as a cross-linker agent that leads to the solidification of the secreted liquid protein adhesive.^21^ The employment of catechol moiety during the adaptive binding of DOPA to various surfaces was further proved using single molecule force measurement.^22^ However, in most cases the synthetic procedures associated with the incorporation of DOPA into antifouling peptides have synthetic complications and require additional steps to avoid these challenges. This leads to an increase in production cost with a low yield of the desired antifouling peptides. In this case polydopamine with adhesive catechol moiety can be considered as a potential alternative and able to mimic the strong adhesion of mussel adhesion proteins (MAPs). Polydopamine (PDA) is a biomimetic polymer produced by the self-polymerization of dopamine (DA) under oxidative and alkaline conditions and is also excreted by many marine organisms.^23,24^ The structure of PDA and the mechanism for its generation are similar to those of melanin, which is caused by the polymerization of l-DOPA. The catechol moiety of PDA can be involved in hydrogen bonding, metal complexation, π–π interactions, and quinhydrone charge-transfer complexation.^25^ Messersmith *et al.* reported that PDA could adhere to different material surfaces, including metals, polymers, and inorganic materials, and this interfacial adhesion property of PDA facilitated the development of the new functionalized materials for various applications.^26^ Compared with other chemical adhesive materials, PDA based coating is more simple as well as economical.^27^ In this context, Rahimipour *et al.* utilized polydopamine to generate functional materials and coatings with antimicrobial property.^28^ Prof. Messersmith reported the PDA modified polymer surfaces comprised of passive and active components exhibit antibacterial property.^29^ But, the development of a multi-layered antifouling coating involving an antifouling polymer with a catechol group as the surface anchor necessitates multistep treatments,^30^ which normally leads to an increase in the production cost. Therefore, the development of an effectual strategy to generate eco-friendly, easy to prepare, biocompatible, and inexpensive coating on different surfaces utilizing PDA is highly demanding to prevent biofouling.

In this present study, we employed a co-assembly technique to attach the adhesive polydopamine nanoparticles on the examined surfaces along with a functionalized antifouling dipeptide (FF–PFB; P1), which exhibits promising antifouling activity. For this purpose, we have functionalized the diphenylalanine (FF) peptide with pentafluoro benzaldehyde (PFB) through the usual condensation reaction, which self-assemble into a thin branched tubular structure. On the other hand, dopamine polymerises into melanin like spherical nanoparticles (PDA NPs). Due to co-assembly, these PDA based nanoparticles with adhesive catechol moiety help the FF–PFB (P1) based self-assembled nanotube to adhere on the desired surface and inhibit the biofilm formation significantly. This co-assembled superstructure obtained from P1 and PDA NPs has (i) a self-assembly unit (FF), (ii) an antifouling unit (PFB) and (iii) adhesive/anchoring moiety (PDA NPs with surface catechol group). These co-assembled superstructures are coated on the desired surfaces (glass and aluminium) by a simple drop-casting method, and these coated surfaces exhibit promising antifouling properties against the tested bacterial strains (*E. coli*, *S. aureus*) and proteins (BSA, lysozyme).

## Results and discussion

### Rational and systematic design of antifouling peptide PFB–Phe–Phe–OMe (P1)

**Scheme 1:**
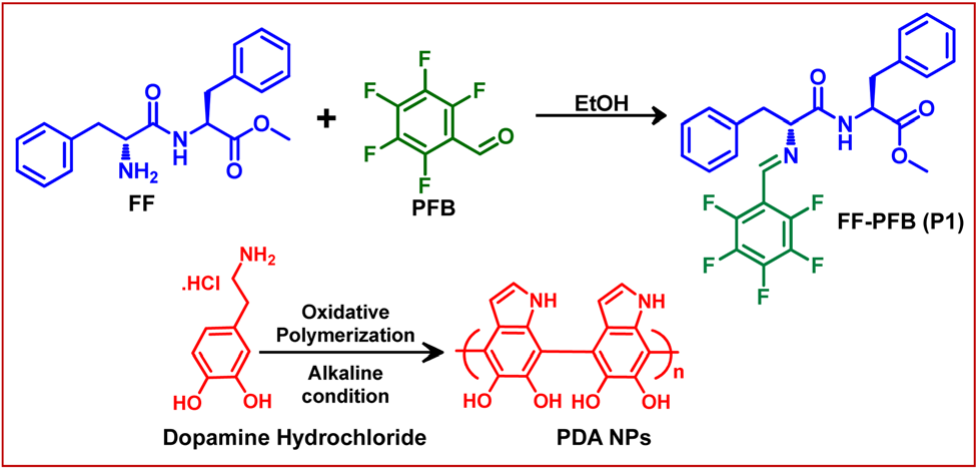
Synthetic methodologies adopted for the synthesis of FF–PFB (P1) and Polydopamine Nanoparticles (PDA NPs).

In this present study, we have designed and synthesized a functionalized dipeptide PFB–Phe–Phe–OMe (FF–PFB) (P1) (Scheme 1). For this purpose, C-terminus-protected aromatic dipeptide diphenylalanine (FF) was coupled with 2,3,4,5,6-penta-fluorobenzaldehyde (PFB) by a simple Schiff base condensation reaction (Scheme 1). The final product was obtained as a pure white solid and characterized by standard analytical techniques. Detailed synthetic procedures are provided in the ESI.† This functionalized dipeptide (P1) is comprised of two basic units: (i) self-assembling unit (FF) and (ii) antifouling unit (PFB). The diphenylalanine (FF) unit, which is the core recognition element of the Alzheimer's disease β-amyloid polypeptide, drives the self-assembly process of this functionalized peptide. It is well known that FF can self-assemble into highly-ordered various supramolecular architectures such as tubes and fibrils under different self-assembly conditions.^31^ In addition to this, so far, FF is the shortest aromatic peptide, which exhibits potential antifouling properties.^32^ The minimalistic structural feature of FF facilitates cost-effective synthesis with high purity, making this a promising unit for developing alternative antimicrobials through proper functionalization with an antibacterial/antifouling group. This functionalization or chemical modifications significantly enhance the efficacy of self-assembled short peptide-based antifouling materials.^32^

**Fig. 1:**
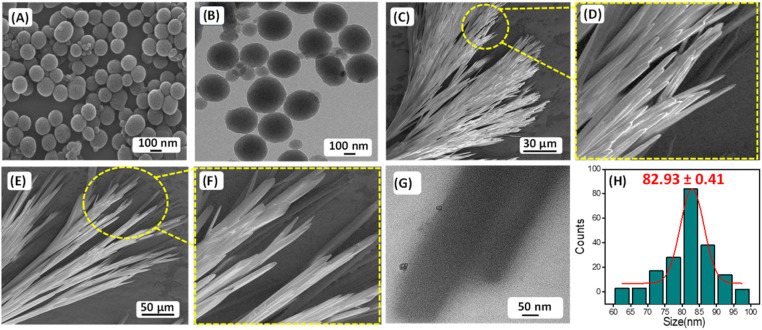
(A) Representative HR-SEM micrograph and (B) HR-TEM micrograph of the PDA NPs. (C)–(F) HR-SEM micrographs of the self-assembled structures formed by P1 in 100% aqueous medium. (G) HR-TEM micrographs of the self-assembled structures formed by P1 in 100% aqueous medium. (H) Size distribution of PDA NPs obtained from HR-SEM analysis.

The presence of carbon–fluorine (–C–F–) bond in the antifouling unit PFB able to form a “Teflon” like coating that will provide better hydrophobic property of the coated surface and can prevent the non-specific adsorption of proteins and bacteria to the desired surfaces. The conjugation of fluorine-substituted aromatic moiety (PFB) with FF-building block was inspired by the earlier report by Reches *et al.*^14^ In their work, they described the presence of one fluorine in the peptide backbone is able to enhance its antifouling property. However, as we discussed earlier, for surface adhesion, an anchoring unit or adhesive unit should be present in the antifouling peptide backbone. Most of the reported antifouling peptides have the unusual amino acid 3,4-dihydroxy-l-phenylalanine (DOPA) with a catechol group as an anchoring unit. As we mentioned previously, the incorporation of DOPA in the peptide backbone has synthetic complications that leads to an increase the production cost of the coated surface. Reches *et al.* described the formation of sticky tubes through co-assembly of diphenylalanine with PDA based melanin like nanoparticles. Simple washing cannot remove sticky tubes attached to the examined surface unless the sample was treated by sonication or dipped in strong acid–alkali (10 M) solutions for five minutes, confirming the adhesive nature of sticky tubes decorated with PDA based nanoparticles.^33^ Therefore, antifouling peptide decorated with PDA-based melanin-like nanoparticles can strongly adhere to the desired surfaces and generate effectual antifouling coating. The PDA nanoparticle also has a reducing property. It can reduce metal ions like Ag^+^, and Au^3+^.^34,35^ But our motive is to employ the co-assembly technique to attach antifouling peptide to the desired surfaces through PDA based adhesive unit, which helps to develop a long-term stable antifouling coating on the desired surfaces. For this purpose, we have synthesized the monodispersed melanin-like polydopamine nanoparticles (PDA NPs) using the previously reported procedure of self-polymerization through aerial oxidation of dopamine hydrochloride under alkaline conditions.^23,24^ The formation of melanin-like polydopamine nanoparticles (PDA NPs) was confirmed by HR-SEM analysis (Fig. 1A), and the average size of the PDA NPs is found to be 82.93 ± 0.41 nm (Fig. 1H). High-resolution transmission electron microscopy (HR-TEM) analysis verified the spherical morphology of the PDA-based nanoparticles with precision (Fig. 1B). PDA is a well-known binder for various surfaces, including several inorganic surfaces like TiO_2_, and SiO_2_. The co-existence of both catechol and amine (lysine) groups help to achieve effective adhesive properties on various surfaces. The choice of dopamine or PDA as a key component for functional coating is based on its structural simplicity and its vital role as a fundamental component of *Mytilus edulis* foot protein 5 (Mefp-5).^26^

### Self-assembly of P1 and co-assembly of P1 and PDA NPs

**Fig. 2:**
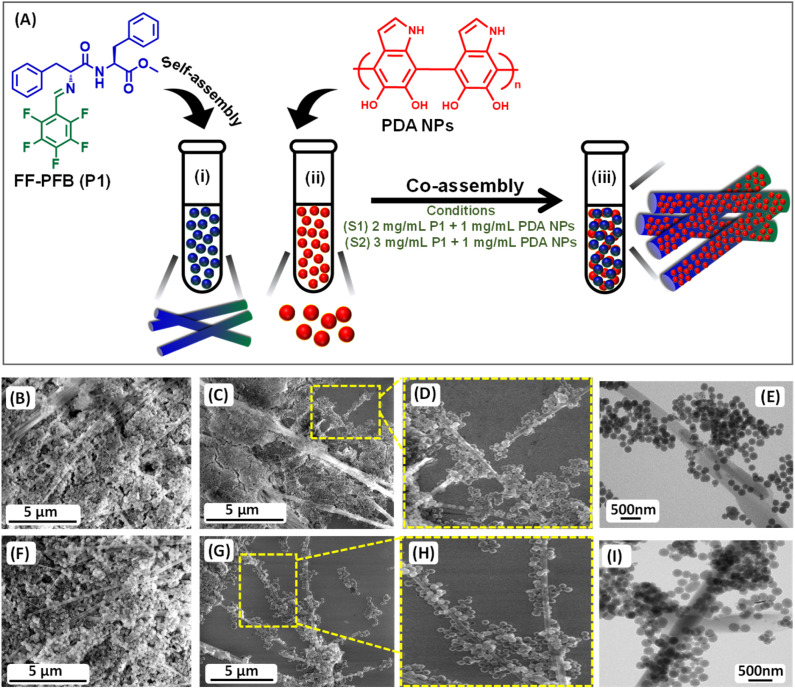
(A) Schematic representation of the co-assembly process in two different conditions: S1 (2 mg mL^−1^P1 + 1 mg mL^−1^ PDA NPs) and S2 (3 mg mL^−1^P1 + 1 mg mL^−1^ PDA NPs). HR-SEM micrographs of the co-assembled superstructures obtained at two different conditions: (B)–(D) S1 and (F)–(H) S2. HR-TEM micrographs of the co-assembled superstructures obtained at two different conditions: (E) S1 and (I) S2.

We have explored the self-assembly property of this newly synthesized peptide P1 (FF–PFB) in an aqueous condition under two different concentrations. To initiate the self-assembly process, we dissolved the P1 peptide in 1,1,1,3,3,3-hexafluoro-2-propanol (HFP) at a concentration of 100 mg mL^−1^ and further diluted with water to achieve the final concentration of 2 and 3 mg mL^−1^. High-resolution scanning electron (HRSEM) microscopy analysis revealed that P1 fabricated into branches of thin tubular-like superstructures in both tested concentrations (Fig. 1C–F). Morphological analysis obtained from HR-SEM is in line with the TEM analysis result (Fig. 1G). After exploring the self-assembly property of the peptide P1, we performed the co-assembly of P1 with PDA NPs (Fig. 2A). For this purpose, we dissolved the peptide P1 in HFP with an initial concentration of 100 mg mL^−1^. Melanine-like PDA NPs were dispersed in DI water by sonication for 30 min to achieve an initial concentration of 1 mg mL^−1^. During this process, the colour of the PDA NPs solution turned into black, confirming the successful dispersion of PDA NPs in DI water. Next, the self-assembly of P1 in the presence of the PDA NPs was performed at two different conditions with two different effective concentrations of P1 peptide (conditions S1 and S2) keeping the concertation of PDA NPs fixed. During co-assembly, the stock solution of P1 in HFP and PDA dispersion in DI was blended together, followed by dilution to get the final desired concentrations. In condition S1, the final effective concentration of the peptide P1 was 2 mg mL^−1^, and in condition S2, the final effective concentration of the peptide P1 was 3 mg mL^−1^. The concentration of PDA NPs in both conditions was kept similar (1 mg mL^−1^). Then, these co-assembled mixtures were incubated for 24 hours at room temperature without any disturbance. HR-SEM analysis was performed to examine the morphology of the fabricated co-assembled superstructures comprised of both P1 and PDA NPs. HR-SEM micrographs clearly displayed the successful stacking of PDA NPs on the thin tubular structures of P1 in both the tested conditions (S1 and S2) (Fig. 2B–D) and (Fig. 2F–H), respectively. Well-defined thin tubular structures decorated with the PDA NPs were formed after 24 hours incubation of the co-assembled mixture under both examined conditions, S1 and S2. HR-TEM analysis further confirmed the morphology of the co-assembled superstructures in both examined conditions (Fig. 2E) for S1 and (Fig. 2I) for S2.

### Coating of examined surfaces with co-assembled superstructures

**Scheme 2:**
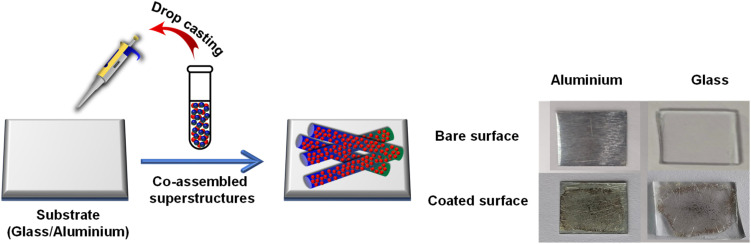
Multifunctional coating formation by drop casting the co-assembled superstructures on examined surfaces (glass and aluminium).

The newly synthesized peptide P1 has both self-assembly and fluorinated antifouling moiety. This antifouling peptide (P1) can be adhered to the different surfaces through co-assembly with PDA NPs with functional adhesive groups, which fabricated sticky tubes on the desired surfaces. Therefore, to explore the potential antifouling property of these co-assembled superstructures, we coated glass and aluminium surfaces with the co-assembled mixtures of two different co-assembly conditions, S1 (2 mg P1 + 1 mg PDA NPs) and S2 (3 mg P1 + 1 mg PDA NPs) by a simple drop-casting method followed by drying in the air under vacuum (Scheme 2). Fabrication of multi-layered antifouling coating comprised of anti-fouling polymer along with suitable surface binder usually involves time-consuming multistep treatments and leads to increased production costs. Considering this, we used simple drop-coating method to coat the examined surfaces (Fig. 7A). This simple technique experiences greater superiority due to its ease of operation without any sophisticated instrumentation, especially for low molecular weight peptides (FF), which leads to a substantial reduction in production costs and expands the scope of commercialization.^36,37^ After the drop casting, the examined surfaces were properly dried under vacuum, followed by 3 times washing with deionized water, which will remove the unbound co-assembled structures. Furthermore, X-ray photoelectron spectroscopy (XPS) was employed to confirm the successful modification of the examined surfaces (glass and aluminium) by the co-assembled superstructures. The XPS analysis revealed a significantly higher atomic concentration of carbon (C), nitrogen (N), and fluorine (F) on the coated surfaces compared to the bare/unmodified surfaces (Fig. S3†). This increase in the carbon and nitrogen content can be attributed to the adhesion of the co-assembled sticky tubes on the examined surfaces, which inherently contain both elements. Furthermore, the detection of fluorine, present in the antifouling moiety of the synthesized peptide P1, confirmed the successful coating of the examined surfaces with the adhesive PDA NPs decorated sticky tubes (Fig. S3†).

**Fig. 3:**
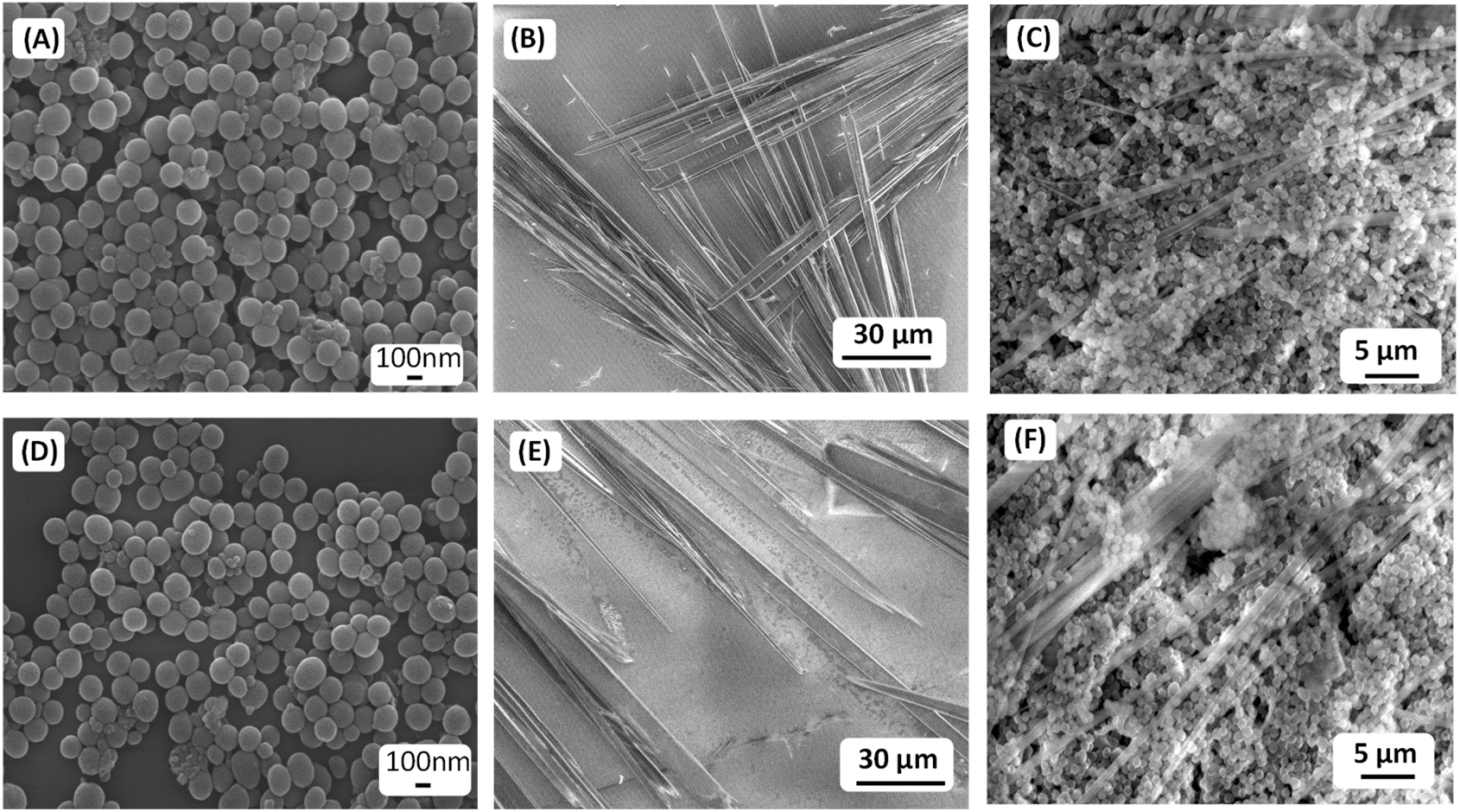
Representative HR-SEM micrographs of the PDA spheres, P1-based branched thin tubular structures, and the co-assembled structures (condition S2) on a glass surface before (A)–(C) and after (D)–(F) water wash.

To evaluate the enhanced adhesive properties or sticky nature of the co-assembled superstructures, both decorated and uncoated (without PDA NPs) tubular structures were placed on a glass substrate and subjected to a simple water-washing test to assess their retention. High-resolution SEM analysis revealed that the uncoated tubular structures were easily washed off from the glass surface by simple water washing, whereas the PDA-NPs decorated tubular structures remained firmly attached to the surface. Only sonication or immersion in strong acid–base (10 M) solutions for five minutes was effective in removing the PDA-NPs decorated tubular structures (Fig. 3). These results suggest that the adhesive property of the co-assembled superstructures, owing to the presence of surface-active functional groups of the PDA NPs.

### UV-vis and FTIR analysis of PDA NPs, self-assembled P1 and co-assembled sticky tubes

**Fig. 4:**
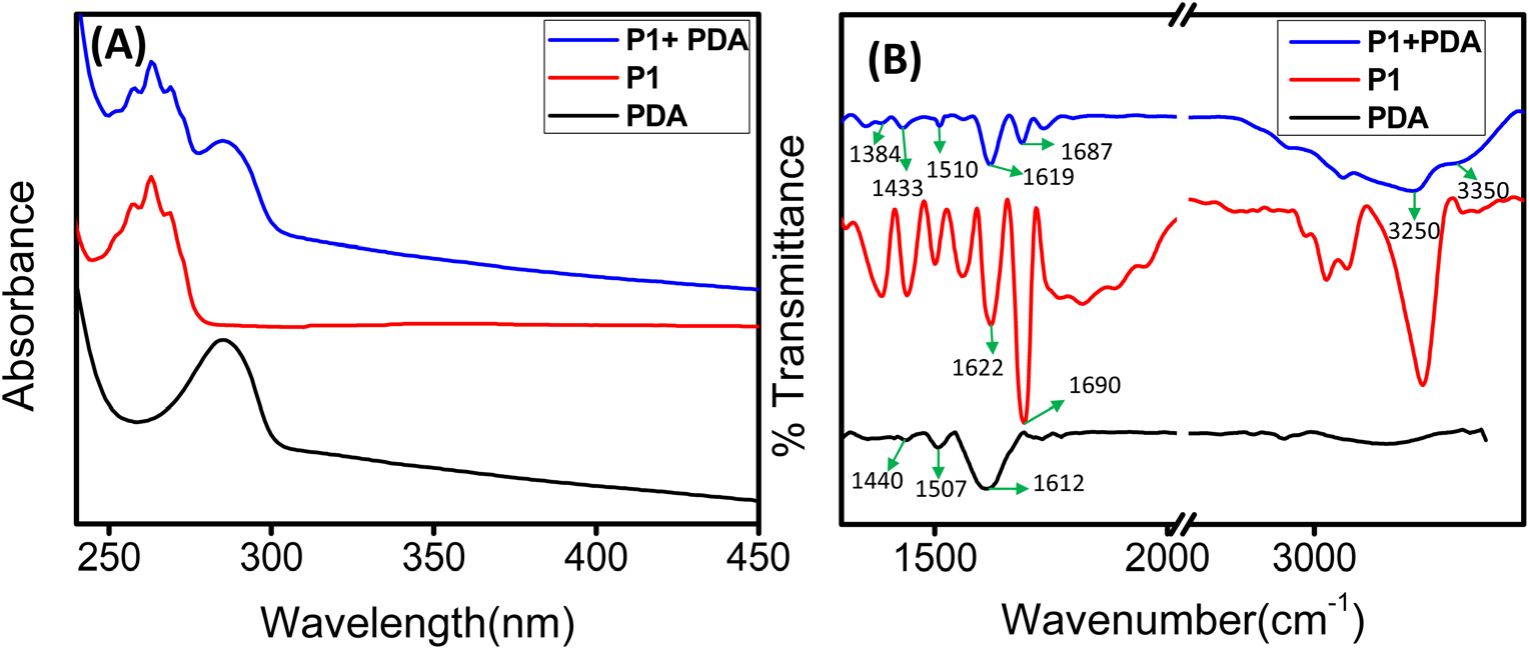
(A) UV-vis absorbance spectra and (B) FT-IR spectra of P1, PDA, and P1 co-assembled with PDA NPs (at 3 mg mL^−1^).

The UV-vis spectroscopic analysis revealed that P1-based tubular structures exhibited an absorption maximum at 263 nm, whereas PDA spheres showed an absorption maximum at 285 nm. The UV-vis spectrum of the co-assembled structures displayed both the peaks (Fig. 4A); however, the intensity corresponds to the absorption peak at 285 nm of PDA spheres diminished and broadened, suggesting that the PDA spheres effectively covered the P1 based tubular structures.^38^ The absorption spectra of P1 is primarily associated with the intramolecular n–π* and π–π* transitions, involving the π-electron cloud of the phenyl ring and the non-bonding electrons of nitrogen, fluorine, and oxygen atoms. In the co-assembled state, the π-electron cloud of the phenyl ring likely engages in π–π stacking interactions with the indole moiety of PDA. At the same time, the non-bonding electron pairs are possibly involved in intermolecular hydrogen bonding with the catechol group of PDA. This could be accounted for the different absorption spectra of the co-assembled state.

Fourier transform infrared (FT-IR) spectroscopy provided critical insights into the chemical composition and secondary structures of the assemblies. The tubular structures formed by the P1 peptide (3 mg mL^−1^) exhibited two distinct absorption peaks at 1622 cm^−1^ and 1690 cm^−1^, characteristic of a β-sheet secondary structure, as documented previously.^31,39^ In contrast, the FT-IR spectrum of the spherical PDA nanoparticles displayed a single peak in the amide I region at 1612 cm^−1^, attributed to the aromatic characteristics of PDA.^38^ Notably, no clear peaks corresponding to a defined secondary structure were observed for the PDA spheres. Characteristic peaks correspond to key functional groups of PDA, such as the amide bond (N–H) shearing band at 1507 cm^−1^, and the aromatic C–C vibrational band at 1440 cm^−1^, were present in the representative FTIR spectrum (Fig. 4B and S4†).^40^ In the co-assembled structures (in 3 mg mL^−1^ of P1), the amide I region revealed two peaks (1687 cm^−1^ and 1619 cm^−1^), closely resemble with bare P1, suggest the existence of its secondary structure in the co-assembled state. Additionally, the characteristic peaks correspond to the functional groups of PDA (1510 cm^−1^, and 1433 cm^−1^) are likely involved in non-covalent interactions with the nanotube walls, and were also present in the FTIR spectrum of the co-assembled structures. This observation indicates a potential interaction between the PDA spheres and the P1-based tubular walls.^26,41,42^ Furthermore, the broader signals with increased intensity at around 3350 cm^−1^ and 3250 cm^−1^ likely arise from N–H/O–H stretching vibrations of surface-active functional groups, further supporting the interaction between the polyphenolic catechol moiety of PDA and the P1-self-assembled nanotubes (Fig. 4B).^43^ A noticeable reduction in the intensity of the FTIR band at 1384 cm^−1^ in the PDA-functionalized P1 nanotubes, compared to the bare P1 nanotubes, could be attributed to π–π stacking and van der Waals interactions between the tubular walls and the aromatic moieties of the PDA molecules.^43^ These findings suggest the strong interactions between the PDA spheres and P1 tubular structures.

### Contact angle and surface roughness measurement

**Fig. 5:**
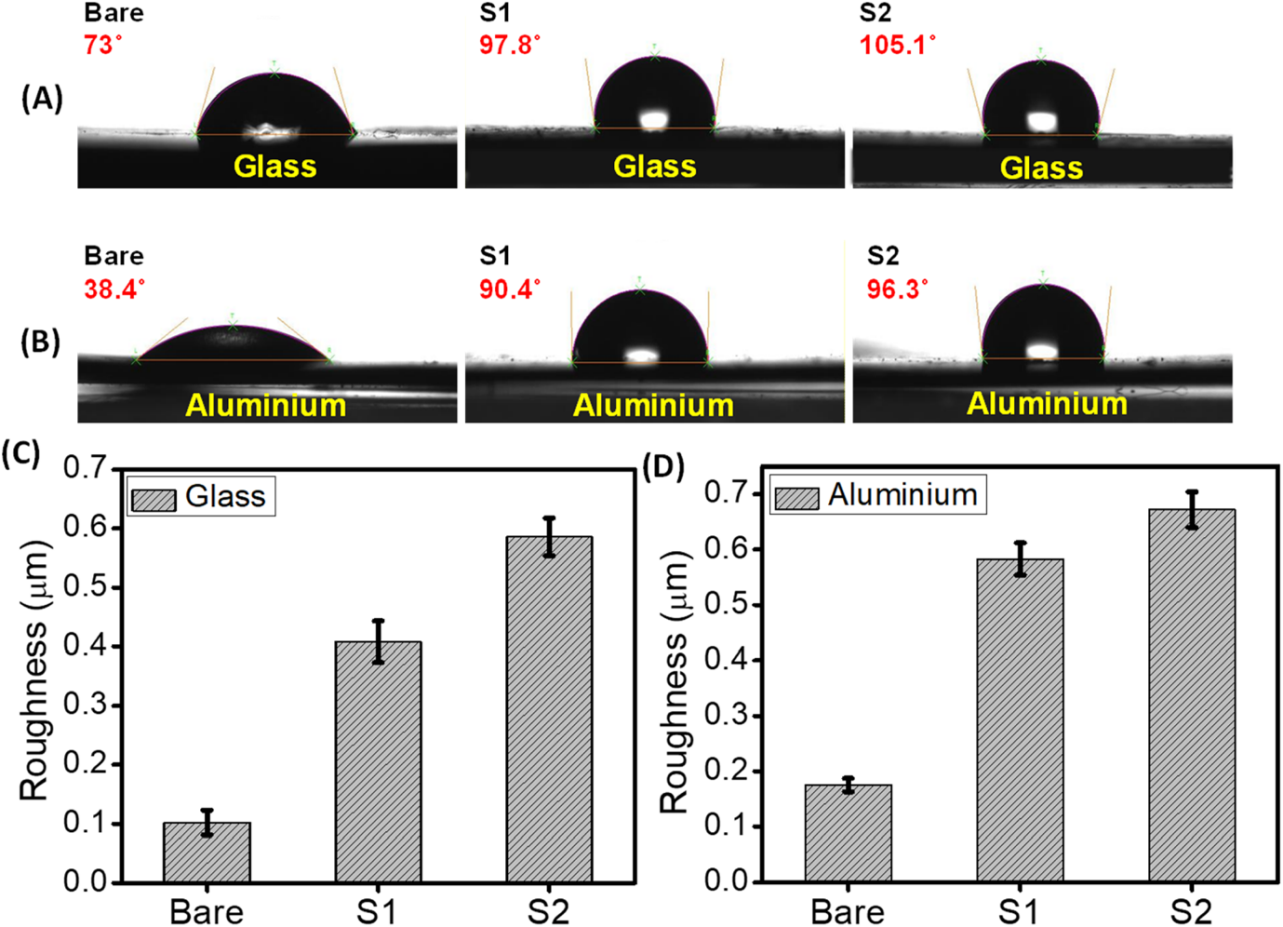
Surface characterization of bare and coated surfaces (glass and aluminium) with co-assembled superstructures in two different conditions, S1 and S2. Contact angle measurements of (A) bare and co-assembled superstructures coated glass surfaces, (B) bare and co-assembled superstructures coated aluminium surfaces. (C) and (D) The bar diagram indicates the surface roughness of bare and coated surfaces. Error bars indicate standard deviation (*n* = 3).

Understanding the interaction of water with these peptides & PDA NPs composite based coated surfaces is an extremely crucial parameter, as both hydrophilic and hydrophobic properties play a significant role in the design of smart antifouling surfaces.^44^ Water contact angle measurements analysis with water droplets revealed that, compared to the bare (uncoated) surfaces, both the coated (glass & aluminium) surfaces displayed a noticeable increase in the hydrophobic property. As shown in Fig. 5A and B, the water contact angle of the coated glass surfaces with different co-assembly conditions (S1 & S2) increased from 73° to 97.8° (with S1 condition) and 105.1° (with S2 condition), respectively. Similarly, in the case of the aluminium surface, the water contact angle increased from 38.4° to 90.4° (with S1 condition) and 96.3° (with S2 condition) (Fig. 5A and B). This increase in hydrophobicity of the examined surfaces coated with co-assembled sticky tubes evidently suggests the formation of “Teflon” like coating. In order to confirm the role of individual component in the hydrophobic properties we conducted additional experiments with proper controls, including individual analyses of P1 and PDA NPs, as well as their co-assembled complexes. The results indicate that PDA NPs exhibit hydrophilic properties (contact angle 55.8°) compared to the bare surface (73°). However, the antifouling peptide P1 with fluorine atoms exhibited hydrophobic properties (82.6°). The synergistic effect between the PDA and P1 enhances the overall hydrophobic properties in its co-assembly state (97.8° and 105.1°) (Fig. S5†).

Roughness is another important parameter, which plays a crucial role in determining the wetting tendency as well as the antifouling performance of a coated surface.^45^ In this regard, we have measured the roughness of the bare (uncoated) and coated surfaces (glass and aluminium) using an optical profilometer. Surface topography analysis displayed that the roughness of both the coated surfaces (glass and aluminium) was significantly increased compared to the bare surfaces without any coating. After successful adhesion of the co-assembled sticky tubes, the roughness of the glass surface increased from 0.102 ± 0.021 μm to 0.408 ± 0.035 μm (coated with S1 condition) and 0.586 ± 0.032 μm (coated with S2 condition) respectively (Fig. 5C). Similarly, in the case of aluminium surfaces, the roughness is increased from 0.175 ± 0.012 μm to 0.583 ± 0.029 μm (coated with S1 condition) and 0.672 ± 0.032 μm (coated with S2 condition) (Fig. 5D). Previous reports also suggest that surface roughness is also responsible for the increased hydrophobic character and antifouling activity of the coated surfaces.^45,46^ These noticeable differences in the hydrophobicity as well as surface roughness is due to the modification of the examined surfaces with the co-assembled sticky tubes comprised of antifouling peptide (P1) and adhesive PDA NPs. The surface topography images obtained from the optical profilometer show the formation of co-assembled superstructures on the examined surfaces with enhanced surface roughness (Fig. S6†).

### Antifouling studies

#### Protein adsorption assay and Kirby–Bauer disk diffusion assay

**Fig. 6:**
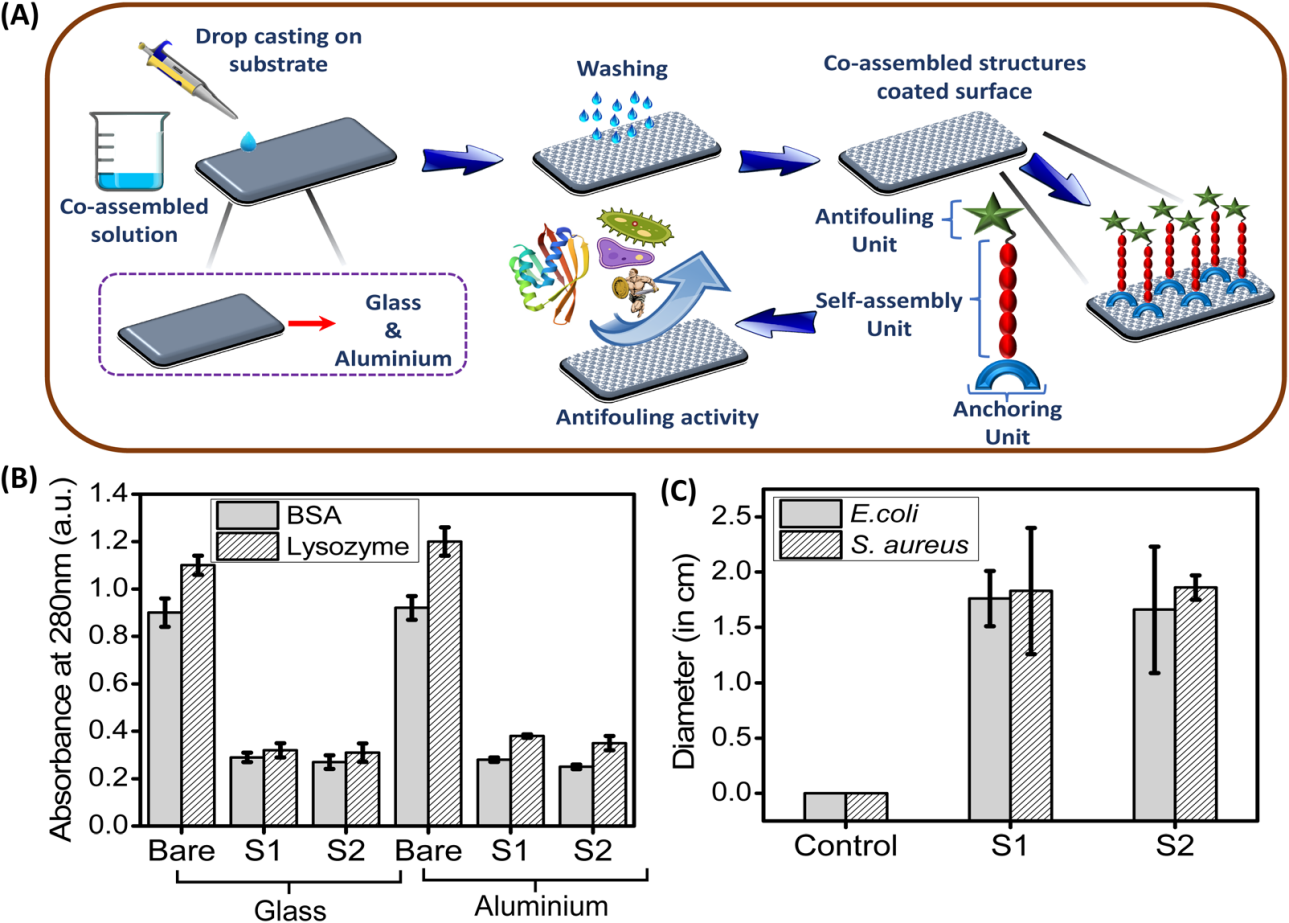
(A) Illustration represents the development of smart antifouling coating on the desired surfaces using the co-assembly of antifouling peptide P1 and adhesive PDA NPs. Evaluation of antifouling activity of the co-assembled superstructures based coated surfaces (glass and aluminium). (B) Adsorbed amounts of BSA (grey) and lysozyme (white) to bare (control) and coated surfaces (glass & aluminium). (C) Zone of inhibition of *E. coli* (grey) and *S. aureus* (white) after 24 hours incubation with two different co-assembly conditions, S1 and S2, as compared to control (DI water). The error bars represent the standard deviation (*n* = 5).

Now, we evaluate the antifouling activity of the coated surfaces with sticky tubes under two different co-assembly conditions (S1 & S2). Initially, during biofilm formation bioorganic materials such as proteins and polysaccharides accumulate on the surfaces, which induces the attachment of bacteria. In this context, at first, we have examined the ability of these glass and aluminium surfaces coated with co-assembled sticky tubes to inhibit the protein adsorption. For this purpose, we have incubated the bare (uncoated) as well as coated surfaces (fabricated under two different co-assembly conditions S1 and S2) in a protein solution of bovine serum albumin (BSA) and lysozyme (Lys). A non-interfering protein assay™ kit was used to determine the adsorbed amounts of BSA and Lys on the bare and coated surfaces, respectively. Fig. 6B clearly shows that higher accumulation of both the proteins BSA and Lys on the bare (uncoated) surfaces compared with the coated glass and aluminium surfaces. A noticeable reduction in protein adsorption on the coated surfaces evidently revealed that the glass and alumina surfaces coated with co-assembled sticky tubes were able to inhibit the protein attachment on the desired surface.

**Fig. 7:**
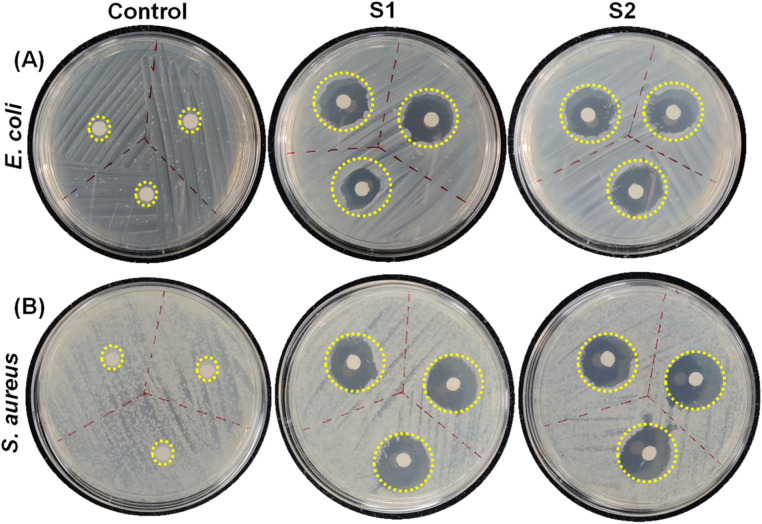
Representative images of the zone of inhibition for (A) *E. coli* and (B) *S. aureus*. The area circled with the yellow dotted line is the area where either a drop of DI water (control) or co-assembled superstructures generated under different co-assembly conditions (S1 & S2) were applied on the agar.

To assess the potential of the co-assembled sticky tube-based superstructures to develop bacteriophobic implant coatings on the examined surfaces (glass, aluminium), our initial investigation mainly focused on their ability to inhibit bacterial growth. We have employed a modified Kirby–Bauer disk diffusion assay to evaluate the ability of the P1 and PDA NPs based co-assembled structures to inhibit bacterial growth against two distinct bacteria Gram (+)ve *Staphylococcus aureus* (*S. aureus*) (MTCC96) and Gram (−)ve *Escherichia coli* (*E. coli*) (MTCC1302) respectively. As illustrated in Fig. 6C and 7, after 16 hours incubation with both the co-assembly conditions (S1 & S2) resulted in a significant zone of inhibitions, averaging ∼1.66 cm (condition S1) and ∼1.76 cm (condition S2) for *E. coli* and ∼1.83 cm (condition S1) ∼1.86 cm (condition S2) for *S. aureus*. This result indicates that the superstructures formed in both co-assembly conditions (S1 & S2) exhibited significant bacteriostatic properties. The control experiments are conducted to confirm the role of individual components in the antibacterial activity. For this we performed zone of inhibition studies using P1, PDA NPs, and the P1–PDA-based co-assembled nanoparticles. The results demonstrated that P1 exhibited significant bacteriostatic properties, while PDA NPs alone showed no measurable antibacterial activity. These findings indicate that the antibacterial properties of the P1–PDA-based co-assembled nanoparticles arise solely from the presence of the antifouling peptide P1 component and not from the PDA NPs (Fig. S7†).

#### Antifouling assay (crystal violet assay)

**Fig. 8:**
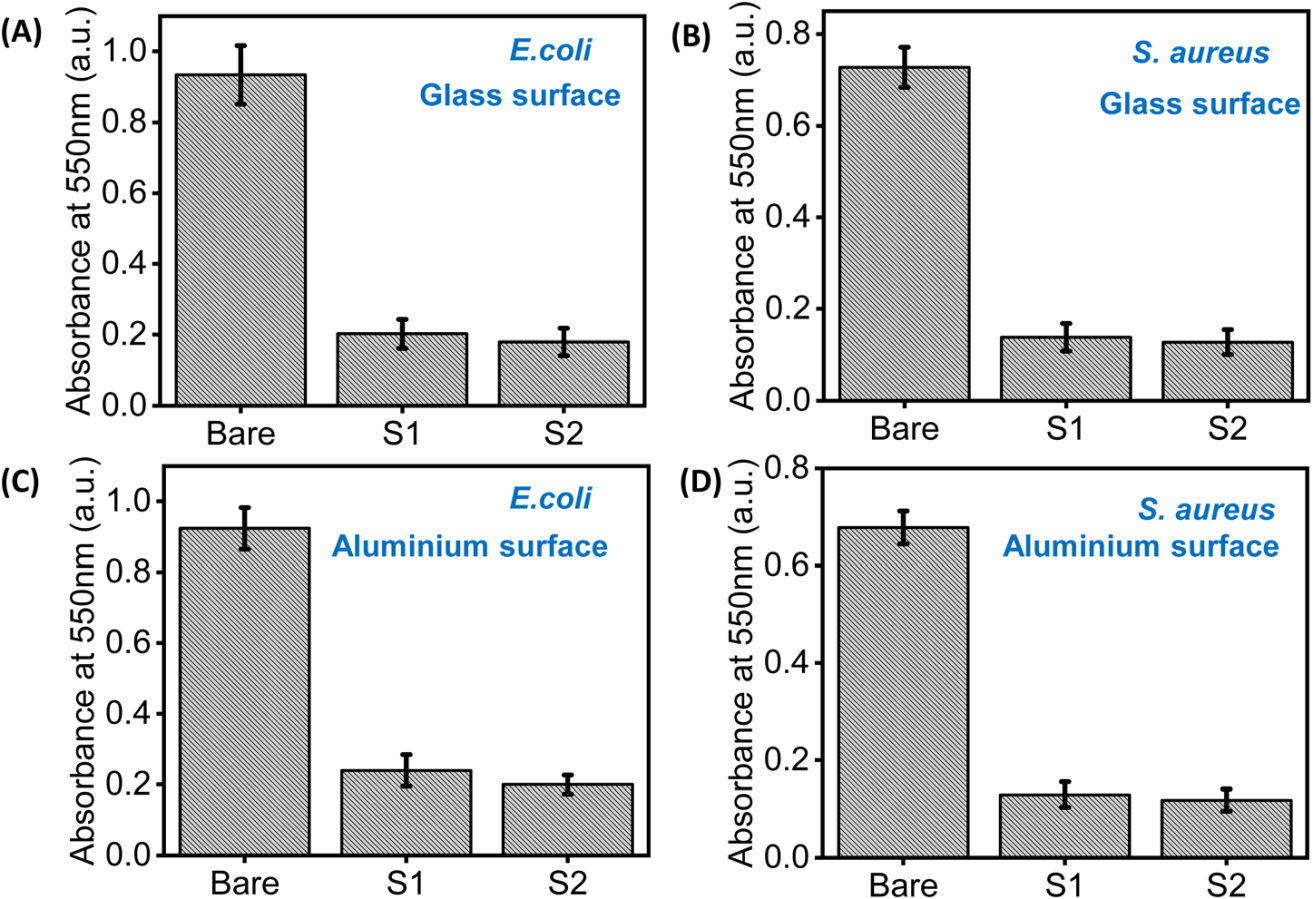
Assessment of antifouling activity of the co-assembled superstructures coated surfaces using crystal violet assay. The OD_550_ value of (A) *E. coli* and (B) *S. aureus* on bare and coated glass surfaces. The OD_550_ value of (C) *E. coli* and (D) *S. aureus* on bare and coated aluminium surfaces.

Our next aim was to evaluate the antifouling ability of the co-assembled sticky tubes based functional coatings against the biofilm formation by *E. coli* and *S. aureus*. The antifouling activity of the bare and coated surfaces was evaluated after 48 hours using the crystal violet assay.^12,47,48^ The significantly lower OD_550_ observed for the coated substrates as compared to the bare (uncoated) substrates indicates that co-assembled sticky tubes exhibited significant antifouling properties (Fig. 8A–D).

#### Crystal violet staining assay

To get a better understanding of the antifouling mechanism, we stained both bare and coated substrates (glass and aluminium) with a 2% crystal violet solution. This experiment was conducted with substrates coated with two different co-assembly conditions (S1 & S2). In Fig. S8,† optical micrographs of the crystal violet stained bare (uncoated) and coated surfaces clearly displayed that the number of bacteria attached to the coated substrates was significantly reduced compared with bare (uncoated) substrates. This experimental result is in well agreement with the bacterial count obtained from the PCM method. Therefore, we can conclude that the glass and aluminium substrates coated with co-assembled PDA based sticky tubes exhibited promising antifouling properties.

#### Toxicity studies

**Fig. 9:**
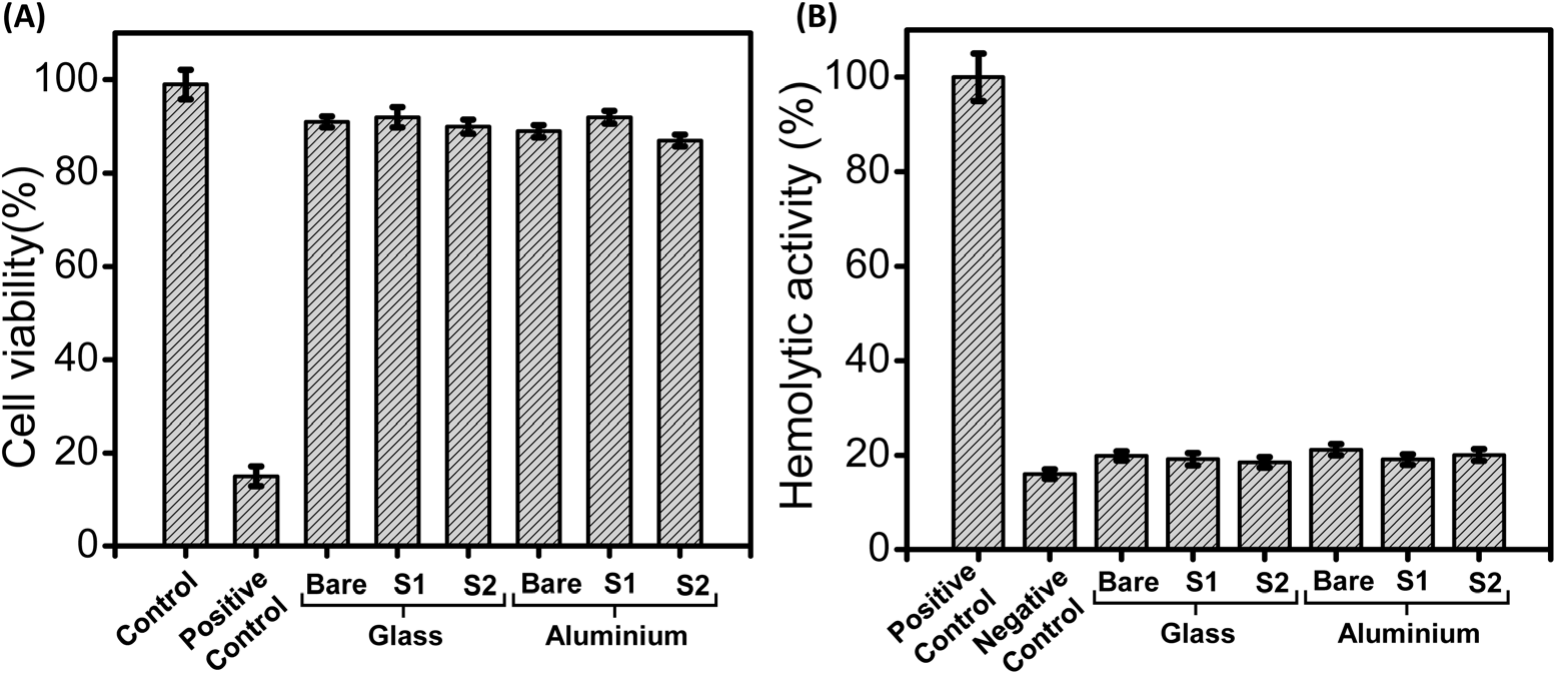
(A) The effect of co-assembled superstructures (formed under conditions S1 and S2) coated surfaces (glass and aluminium) on the viability of HEK293 cells as measured by MTT assay. (B) The hemolytic activity of the co-assembled superstructures (formed under conditions S1 and S2) coated surfaces (glass and aluminium). The co-assembled superstructures coated surfaces did not show any hemolytic activity.

In order to employ this co-assembled PDA-based sticky tubes coated antifouling surfaces for potential biomedical applications, we have also examined their cytotoxicity to human cells by conventional MTT assay^49^ and haemolytic assay.^50,51^ The eventual cytotoxicity of the co-assembled superstructures coated on both glass and aluminium surfaces was tested in human embryonic kidney cells (HEK293). Cell proliferation was estimated after 12 hours incubation following the standard protocol.^51^ As shown in Fig. 9A, there were no substantial differences in the cell proliferation observed after incubation with sticky tubes coated glass and aluminium surfaces evidently displayed their significant biocompatibility (∼85–90%). Additionally, we have performed the MTT assay for the co-assembled superstructures (S1, S2) coated on both surfaces, P1 and PDA with a duration of 48 hours. The results indicate that co-assembled superstructures, P1 and PDA do not exert any significant cytotoxic effects, demonstrating their biocompatibility after 48 hours (Fig. S9†). In addition, hemolytic activity was also evaluated by spectroscopic measurements of the amount of hemoglobin released from erythrocytes following treatment with the co-assembled superstructures coated glass and aluminium surfaces. After incubation with the co-assembled superstructures modified surfaces, over 83% of erythrocytes remained intact and undisrupted, as represented in Fig. 9B. These results clearly demonstrate that the co-assembled superstructures obtained through the co-assembly of P1 and PDA NPs are biocompatible and non-hemolytic.

## Conclusion

In conclusion, our study has described the formation of sticky tubes through the co-assembly of antifouling peptide P1 and PDA NPS with adhesive catechol moiety. Glass and aluminium surfaces coated with these co-assembled sticky tubes (formed under two different co-assembly conditions S1 and S2) using simple drop casting method. The strategic combination of these components is able to form stable, biocompatible, and multifunctional coatings on desired surfaces (glass and aluminium) with superior antifouling properties. Our investigation exhibited the antifouling efficacy against two common bacterial strains, *Escherichia coli* (*E. coli*) and *Staphylococcus aureus* (*S. aureus*). The coated surfaces effectively inhibited the bacterial adhesion, and displayed their potential as promising materials for various biomedical and industrial applications. The employment of the functional antifouling peptide P1 in the formation of a biocompatible coating that actively repels bacterial and protein attachment. Moreover, the adhesive properties of PDA NPs with catechol moiety played a pivotal role in enhancing the stability and longevity of the coatings. The synergistic effects observed in the co-assembled superstructures underline the significance of a well-designed combination of antifouling and adhesive components for the development of smart antifouling materials. This co-assembled superstructures-based antifouling coating holds significant promise for broader applications, including medical devices, marine coatings, and water treatment systems. Future research in this area could further explore the optimization of the co-assembly process using two different functional components and investigate the performance of these multifunctional superstructures under diverse environmental conditions.

## Experimental section

### Materials and methods

All chemicals and solvents are commercially available and were used as received without further purification. l-Phenyl alanine, *N*,*N*-dicyclohexylcarbodiimide (DCC), 1-hydroxybenzotriazole (HOBt), BOC anhydride, trimethylchlorosilane (TMSCl), 1,1,1,3,3,3-hexafluoro-2-propanol (HFP), *N*,*N*-diisopropylethylamine, dopamine hydrochloride and triethyl amine were purchased from Sisco Research Laboratories Pvt. Ltd (SRL, India). Potassium hydrogen sulfate, HCl, sodium chloride, sodium sulfate, sodium hydroxide, and sodium carbonate were purchased from Finar Chemicals Pvt. Ltd (India). Nutrient agar and tryptone soya broth (TSB) were purchased from Himedia Laboratories. 9-Well plates, 12-well plates, 90 mm Petri dish, inoculation needle, microcentrifuge tube (2 mL), and Falcon conical centrifuge tube were purchased from Tarson. Glass slides, phosphate buffer saline (PBS), crystal violet, ethanol, and isopropyl alcohol were purchased from Sisco Research Laboratories (SRL) Pvt. Ltd, India.

### Preparation of substrates

Glass and aluminium substrates of 1 cm^2^ were first washed thoroughly for 2 minutes each with DI water, acetone, and isopropyl alcohol, followed by 100% ethanol before autoclaving them. Two different co-assembled solutions were dropped on to the substrates, dried properly, washed with deionized water, and kept in a clean Petri dish, and incubated for 4 hours at RT. The slides were rinsed and used.

### High-resolution scanning electron microscopy (HR-SEM)

A 10 μL drop of a self-assembled/co-assembled solution of P1 and PDA NPs was placed on a glass coverslip and allowed to dry at RT. Then, the SEM analysis was performed using a high-resolution scanning electron microscope (Thermoscientific, Apreo S) operating at 18 kV.

### Contact angle measurement

Contact angle measurements were carried out using a substrate-based analyzer at the solid/water interface (model no.: HO-IAD-CAM-01A, Holmarc, Opto-Mechatronics Pvt. Ltd). Each experimental measurement consisted of three repeats, and the reported angles were averaged.

### Surface roughness measurement

The surface roughness of the co-assembled superstructures obtained at two different conditions (S1 & S2) was measured using an optical profilometer (KLA Tencor-US, model: MICROXAM 800).

### Bacterial growth

A single colony of each bacterial strain (*E. coli* and *S. aureus*) was immersed in 20 mL of TSB in a loosely capped tube. Bacteria were grown at 37 °C with agitation (120 rpm) for overnight. Then, bacteria were centrifuged, washed with PBS (3 times), and resuspended in TSB to a concentration of 10^7^ CFU mL^−1^. The concentration of bacteria was determined by measuring the optical density (OD) at 600 nm (UV-Vis Spectrophotometer, Shimadzu, Kyoto, Japan).

### Protein adsorption assay

50 mL of a single protein solution of BSA and lysozyme (150 mM in PBS) was applied onto the co-assembled structures coated substrates in a Petri dish. The plate was placed in a humidified incubator at 37 °C for 3 hours. The substrates were then rinsed 3 times with PBS (pH = 7.43, 10 mM, 150 mM NaCl) and transferred into test tubes with 1 mL of 1.0% (w/w) SDS. The samples were shaken for 60 minutes and sonicated for 20 minutes at room temperature to detach the adsorbed proteins. Protein concentrations in the SDS solution were determined using the non-interfering protein assay (Merck, Millipore) according to the manufacturer's instructions using a microplate reader at 280 nm (Biorad). All measurements were performed five times and averaged.

### Anti-fouling assay (crystal violet assay)

To evaluate the efficient antifouling activity of the co-assembled structures, both coated and bare glass and aluminium surfaces were incubated in a medium containing *E. coli* and *S. aureus* at 37 °C for 48 hours. After incubation, the bare and coated surfaces were washed three times with 1× PBS to remove planktonic cells. Subsequently, the surfaces were stained with a 1% crystal violet solution for 20 minutes to visualize adherent biofilms. Excess stain was removed by rinsing the surfaces with 1× PBS, followed by air drying. The bound dye was then solubilized using 30% acetic acid, and the optical density (OD) was measured at 550 nm to quantify biofilm formation.^12,47,48^

#### Zone of inhibition study

A single colony (either *E. coli* or *S. aureus*) was immersed in 3 mL TSB media. Then, the bacterial suspension was spread over agar plates homogeneously using a sterile swab. 20 μL drops of co-assembled solutions at two different conditions S1 (2 mg mL^−1^P1 + 1 mg mL^−1^ PDA NPs) and S2 (3 mg mL^−1^P1 + 1 mg mL^−1^ PDA NPs) were dropped at different spots on the agar. A drop of water was used as a control. The plates were then incubated overnight at 37 °C.

### Crystal violet assay

Briefly, after incubation with the bacteria as mentioned above, the Ag–PDA-coated substrates were gently rinsed 3 times and stained with 2% crystal violet at room temperature for 20 minutes. The stained samples were rinsed 3–4 times with water and left to dry in air. Then, the substrates were visualized under optical microscopy (Leica DM6 Fluorescent Microscope with Cryostat) to evaluate the amount of bacteria adhered to the substrate.

### Evaluating the *in vitro* toxicity of the co-assembled structures

#### Hemolysis assay

A hemolysis assay was conducted to assess the hemolytic activity of ultra-short peptide-coated surfaces. Sheep blood (Labline Trading Co., ref.: DSFB-200ML, lot: 0118) was taken in a centrifuge tube and centrifuged at 1000*g* for 5 minutes at 4 °C. The supernatant was discarded, and the pellet was washed twice with 1× phosphate-buffered saline (PBS) (10× PBS, Himedia, catalogue no.: ML023) and resuspended in 1× PBS. Freshly prepared blood was then treated with the peptide-coated surfaces in a microcentrifuge tube. The samples were incubated at 37 °C for 2 hours. After incubation, the tubes were centrifuged at 1000*g* for 5 minutes at 4 °C. The supernatant was collected, and hemolytic activity was measured by spectroscopic analysis (OD_405_) of the released hemoglobin using a BioTek Epoch 2 Reader – Microplate Spectrophotometer. 1% Triton X-100 (Sigma Aldrich, catalogue no.: T8787-60ML, lot # SLCJ6163) served as the positive control, while 1× PBS was used as the negative control.^50^

#### MTT assay

An MTT assay was conducted to evaluate the cytotoxicity of ultra-short peptide-coated surfaces. HEK293 cells (from National Centre For Cell Science, Pune) were cultured in Dulbecco's Modified Eagle Medium (DMEM) (Sigma, catalogue no.: D777-10X1L, lot # SLBZ1832) with 10% fetal bovine serum (Gibco, ref. 10270-106, lot # 42 G3099K) and 1% penicillin–streptomycin (Invitrogen, catalogue no.: 15140122) at pH 7.4 in a 5% CO_2_ humidified atmosphere at 37 °C. Approximately 0.2 × 10^6^ HEK293 cells were seeded in a 12-well plate and incubated overnight under the same conditions. Upon reaching 70–80% confluency, cells were treated with the peptide-coated surfaces for 12 hours. The MTT assay was performed using the EZcount MTT Cell Assay Kit (Himedia, product code: CCK003), and absorbance was measured at 570 nm using a BioTek Epoch Microplate Spectrophotometer (Agilent). Untreated cells served as the cell control. Cell viability (%) for each sample was plotted. 2% Triton X-100 (Sigma Aldrich, catalogue no.: T8787-60 ML, lot # SLCJ6163) was the positive control, and PBS was the negative control.^49^

## Data availability

Characterization data along with further supporting data referenced in the manuscript are available in the ESI.†

## Author contributions

Conceptualization and methodology, P. D. and A. S.; acquisition of data, S. S., S. N., A. H.; analysis, investigation, and interpretation of data, S. S., S. N., A. H., O. M., A. S., and P. D.; writing – original draft and writing – review and editing, S. S., S. N., A. H., O. M., and P. D.; supervision, O. M., A. S., and P. D.; S. S., and S. N. contributed equally.

## Conflicts of interest

There are no conflicts of interest to declare.

## Supplementary Material

RA-015-D4RA08342C-s001
