# Supplementary material for: Sticky tubes co-assembled by functionalised diphenylalanine and polydopamine nanoparticles form biocompatible antifouling coating
Source: RSC Adv. 2025 Feb 5;15(5):3672–85. doi: 10.1039/d4ra08342c (PMC11795260; doi:10.1039/d4ra08342c)
Supplement: RA-015-D4RA08342C-s001 [file RA-015-D4RA08342C-s001.pdf]

Supplementary Information (SI) for RSC Advances.
This journal is © The Royal Society of Chemistry 2025

Supplementary Information

Sticky Tubes Co-assembled by Functionalised Diphenylalanine and polydopamine nanoparticles form Biocompatible antifouling coating

Subramaniyam Sivagnanam[#a], Suman Nayak[#a], Arpita Halder[b], Oindrilla Mukherjee[b], Abhijit Saha*[a] and Priyadip Das*[a]

[a]Department of Chemistry, SRM Institute of Science and Technology, SRM Nagar, Potheri, Kattankulathur, Tamil Nadu-603203

[b]Department of Biotechnology, National Institute of Technology Durgapur, West Bengal, 713209, India

[#]equal contribution

*Corresponding author

Priyadip Das, E-mail: priyadipcsmcri@gmail.com , priyadip@srmist.edu.in

Abhijit Saha, E-mail: abhijits@srmist.edu.in

Peptide synthesis

Synthesis of H_2N-Phe-Phe-OMe (A4)

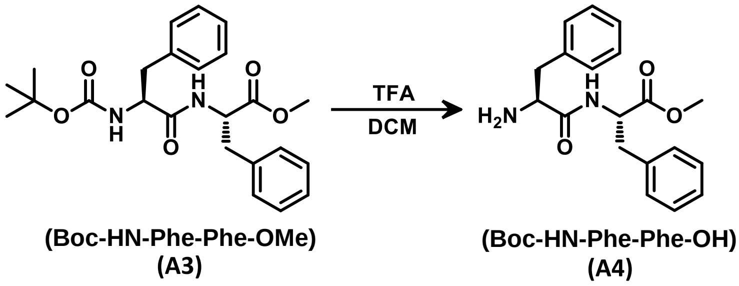

Scheme S1: Synthetic methodologies adopted for the synthesis of H_2N-Phe-Phe-OMe.

Boc-Phe-Phe-OMe was synthesized according to our previous report.[1] 2 g (4.68 mmol) of Boc-Phe-Phe-OMe was dissolved in 30 mL of DCM in an ice bath. Then, 8 mL of TFA was added drop by drop and stirred for 2h. The progress of the reaction was monitored by TLC. After the reaction was completed, all solvents were evaporated in a rotary evaporator. The product was then dissolved in water, neutralized with $NaHCO_3$ solution, extracted with ethyl acetate, dried over anhydrous sodium sulfate and evaporated by rotary evaporator to obtain an oily product, which was immediately used for the next reaction. Yield: 1.42 g (4.35 mmol, 93.42%) (Scheme S1).

Synthesis of PFB-Phe-Phe-OMe (A4)

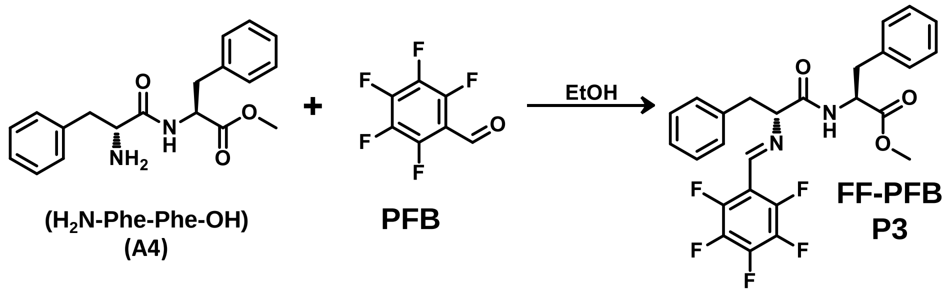

Scheme S2: Synthetic methodologies adopted for the synthesis of PFB-Phe-Phe-OMe.

100 mg of H_2N-Phe-Phe-OMe dissolved in 10 ml of EtOH and 45 μL of PFB was added to the mixture. Then, this solution was refluxed at 82˚C for 8 hrs and the progress of the reaction was monitored by TLC. After the reaction was completed, the remaining solvent was evaporated and washed with n-hexane and cold EtOH. The product obtained was dried and obtained as pure solid. Yield: 125.23 mg (81%). ^{1}H NMR (500 MHz, DMSO-d_6) δ 9.02 (s, 1H, CH=N), 8.15 (s, 1H, NH), 7.34-7.31 (m, 3H, ArH), 7.30-7.26 (m, 4H, ArH), 7.24-7.23 (m, 3H, ArH), 4.60-4.56 (q, J=7.35, 1H, CαH), 4.06-4.03 (t, 1H, CαH), 3.62 (s, 3H, OMe), 3.13-3.05 (m, 2H, CβH), 3.00-2.91 (m, 2H, CβH). HRMS MS (m/z): $[M+H]^+$= 505.1551 (calculated); 505.1544 (observed) (Scheme S2).

Synthesis of Poly dopamine nanoparticles (PDA NPs)

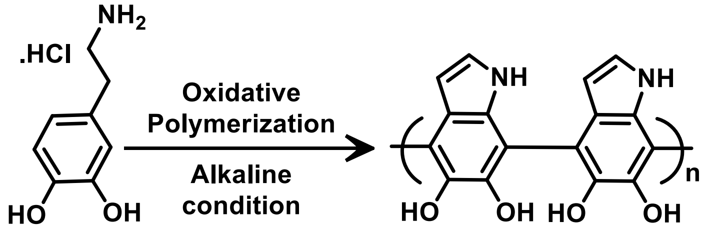

Scheme S3: Synthetic methodologies adopted for the synthesis of polydopamine nanoparticles (PDA NPs).

180 mg of dopamine hydrochloride was dissolved in 90 mL of DI water under vigorous stirring. 760μL of 1N NaOH solution was added at 50˚C, with the color of the solution turning to pale yellow as soon as NaOH was added and gradually changing to dark brown. After aging (5 hrs), nanoparticles were retrieved from centrifugation and washed with DI water several times. Melanine like nanoparticles were then selected as a dispersed solution after all large-size materials were removed by slow-speed centrifugation (4000 rpm).

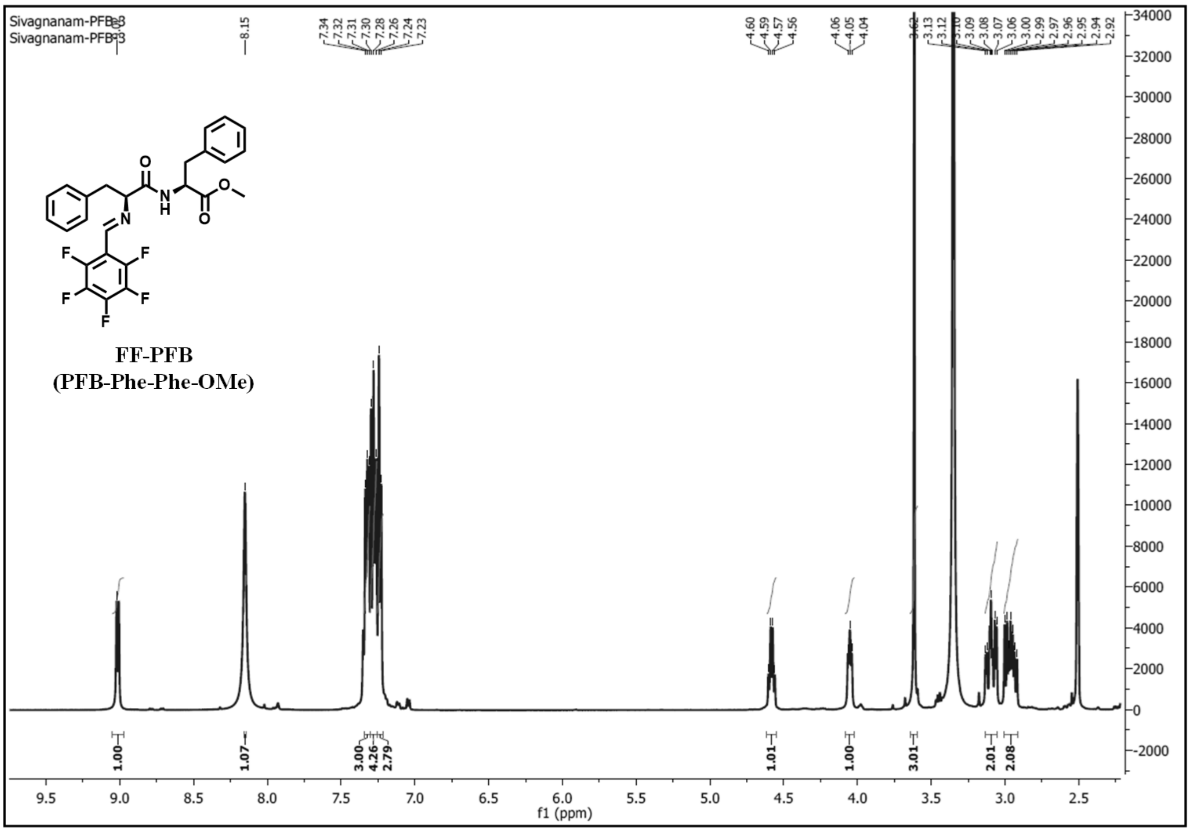

Figure S1. ^{1}H NMR (DMSO-d_6, 500 MHz, δppm) of PFB-Phe-Phe-OMe (**FF-PFB**).

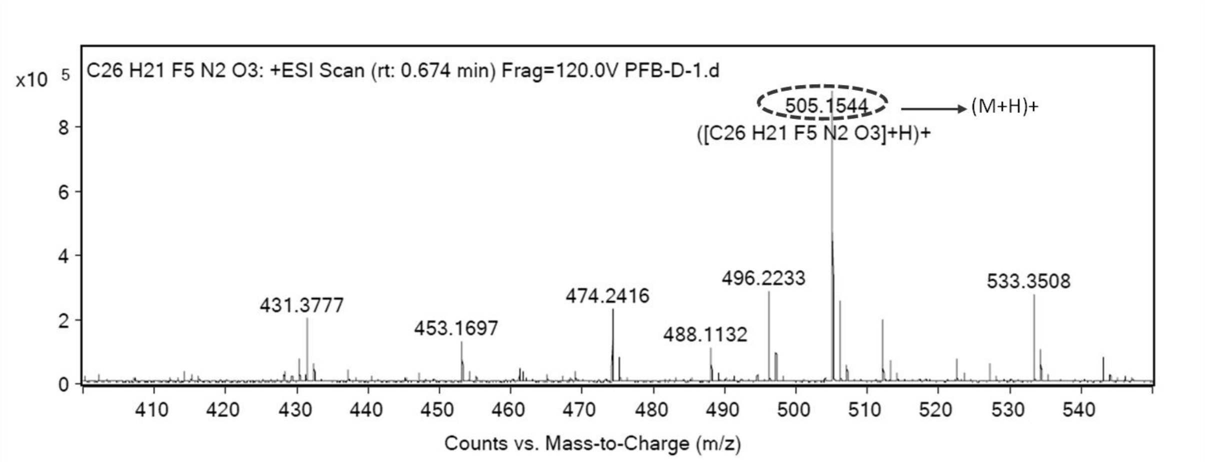

Figure S2. High-Resolution Mass spectra of PFB-Phe-Phe-OMe (**FF-PFB**).

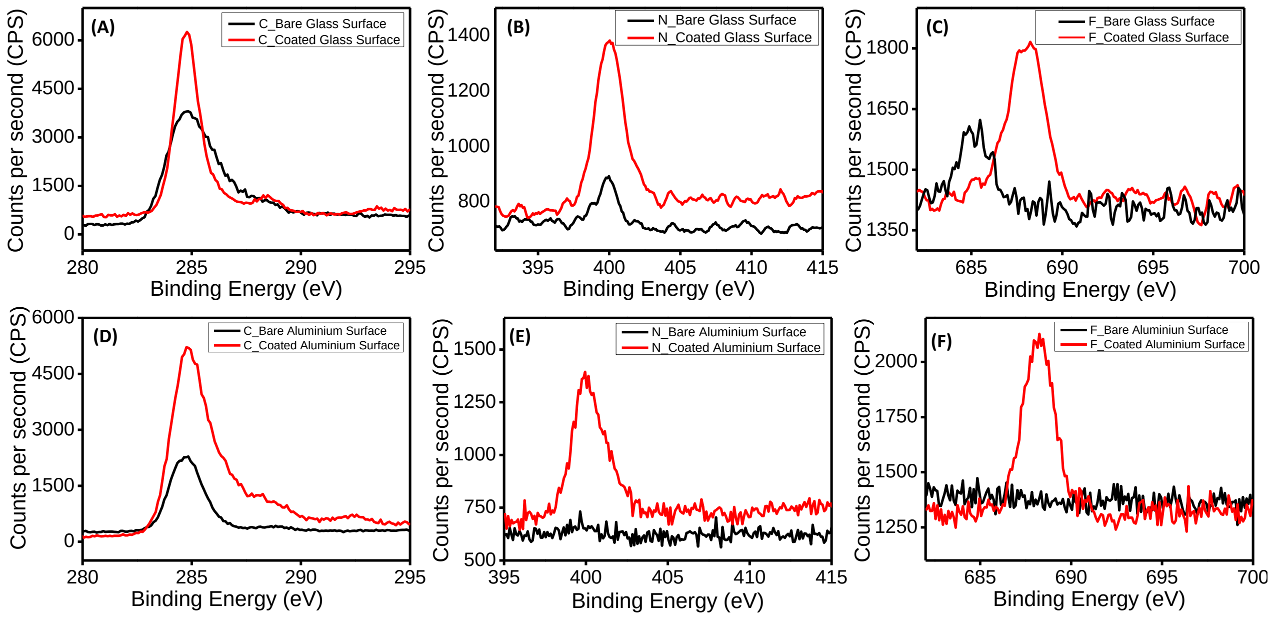

Figure S3: XPS analysis of Bare and Coated glass surface:(A) carbon (B) nitrogen, (C) fluorine, and Bare and Coated aluminium surface:(D) carbon (E) nitrogen, (F) fluorine.

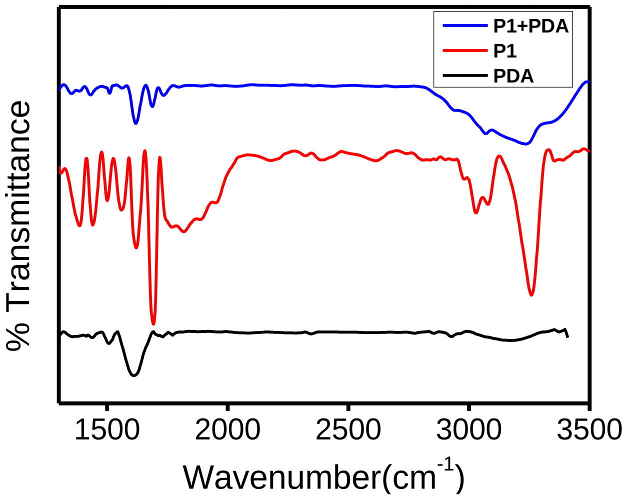

Figure S4: The full range of FT-IR spectra of **P1**, PDA, and **P1** co-assembled with PDA NPs (at 3mg/mL).

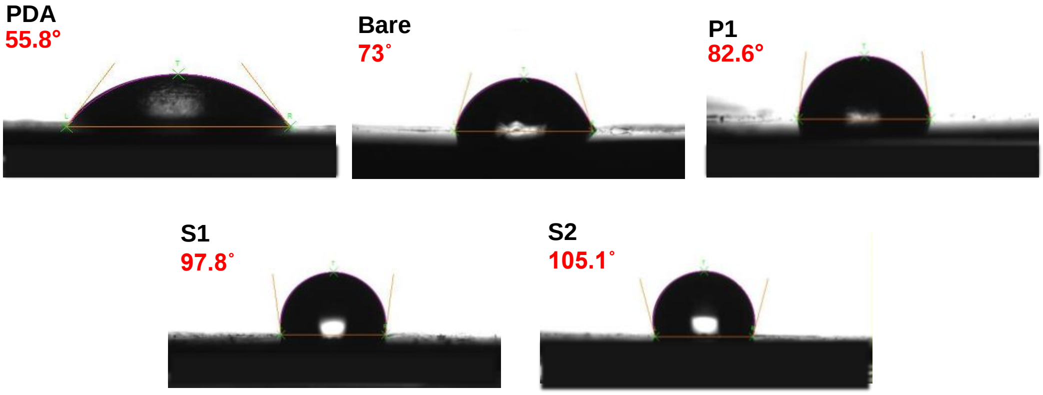

Figure S5: Contact angle measurements of bare, PDA, **P1**, and **P1** co-assembled with PDA superstructures coated glass surfaces.

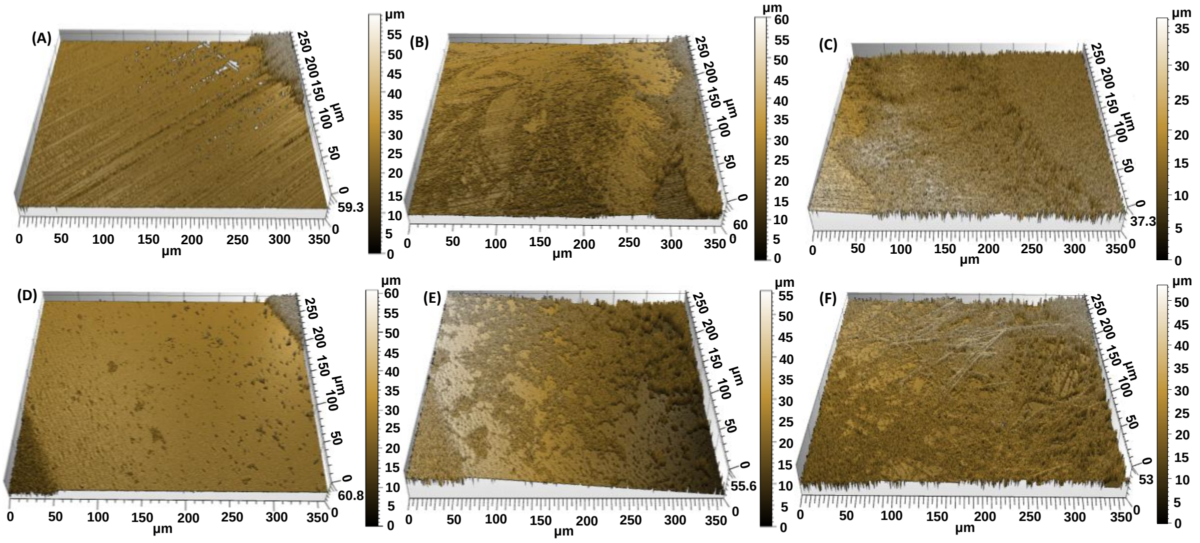

Figure S6: Surface topography observed by an optical profilometer. (A) surface topography of bare glass surface and glass surfaces coated with co-assembled superstructures in two different co-assembly conditions (B) **S1** and (C) **S2**. (D) Surface topography of bare aluminium surface and aluminium surfaces coated with co-assembled superstructures in two different co-assembly conditions (E) **S1** and (F) **S2**.

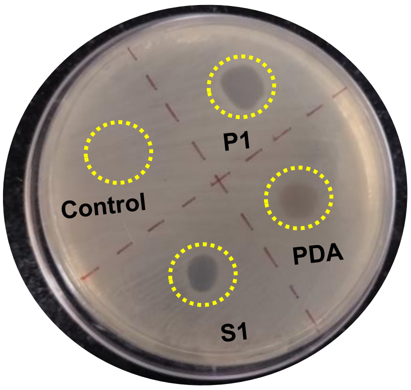

Figure S7: Representative images of the Zone of inhibition for control, **P1**, PDA, and co-assembled superstructures. The area circled with the yellow dotted line is the area where either a drop of DI water (Control) or samples were applied on the agar.

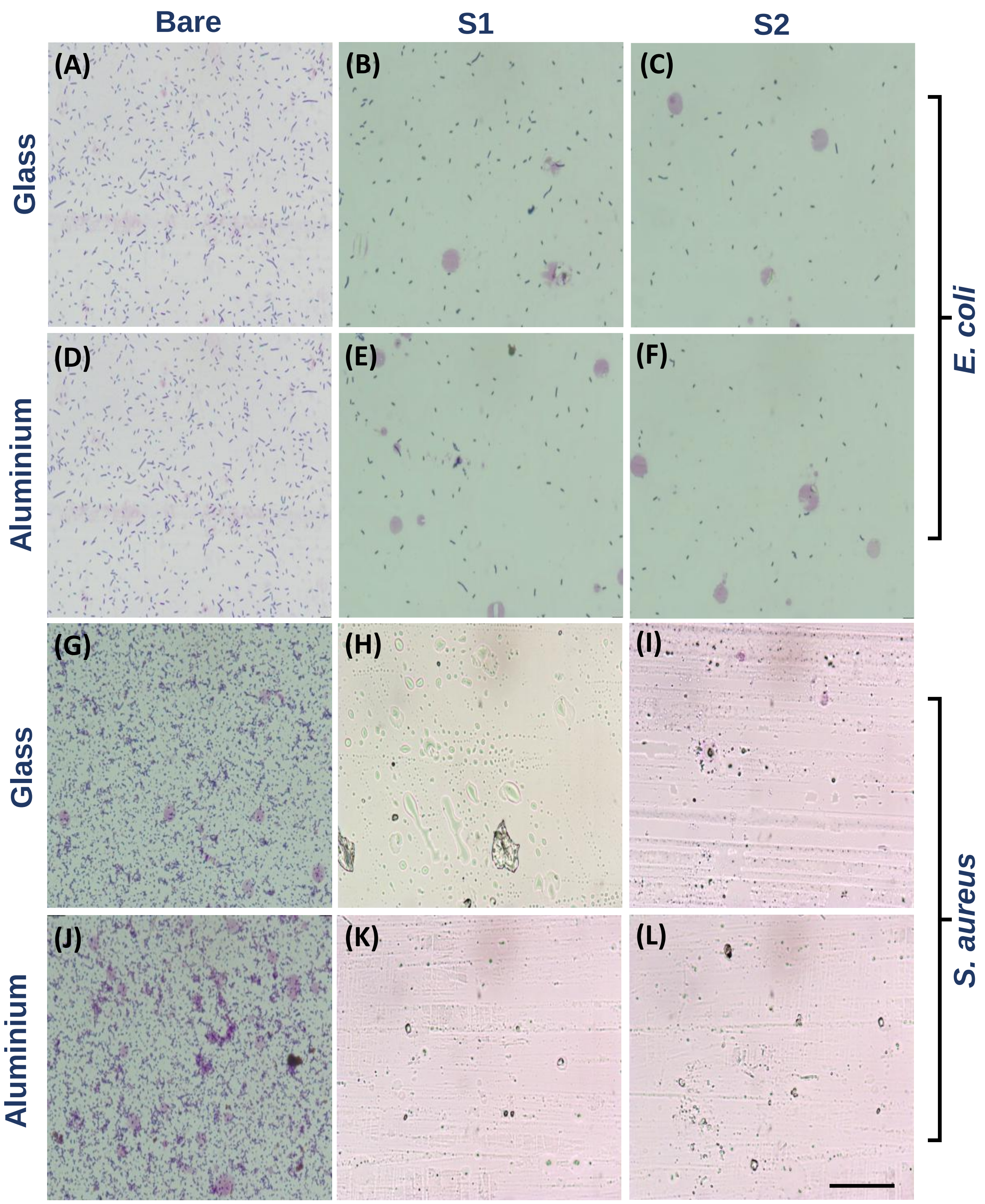

Figure 8: Optical microscopic images of surfaces (glass and aluminium) coated with co-assembled superstructures obtained from two different co-assembly conditions **S1** and **S2**: Crystal violet staining of *E. coli* grown on (A-C) glass and (D-F) aluminium surfaces. Crystal violet staining of *S. aureus* grown on (G-I) glass and (J-L) aluminium surfaces. (Scale bar 10μm).

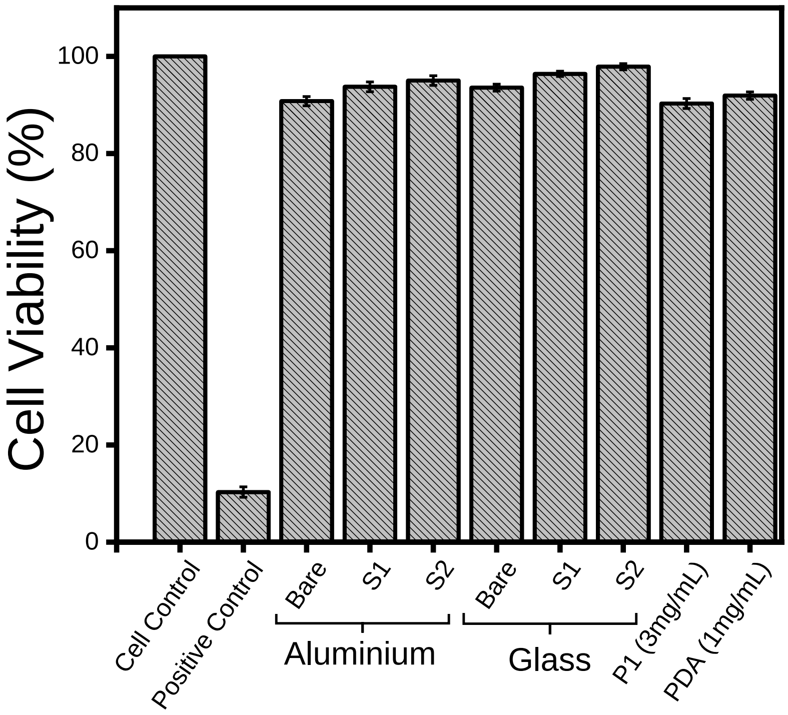

Figure S9: The effect of co-assembled superstructures (formed under conditions **S1** and **S2**) coated surfaces (glass and aluminium), **P1**, and PDA on the viability of HEK293 cells as measured by MTT assay.

References

1 S. Sivagnanam, A. Arul, S. Ghosh, A. Dey, S. Ghorai and P. Das, Concentration-dependent fabrication of short-peptide-based different self-assembled nanostructures with various morphologies and intracellular delivery property, *Mater. Chem. Front.*, 2019, **3**, 2110–2119.
